# Comparison of Standardized Methods for Determining the Diffusion Coefficient of Chloride in Concrete with Thermodynamic Model of Migration

**DOI:** 10.3390/ma16020637

**Published:** 2023-01-09

**Authors:** Zofia Szweda, Jacek Gołaszewski, Pratanu Ghosh, Petr Lehner, Petr Konečný

**Affiliations:** 1 Department of Building Structures, Faculty of Civil Engineering, Silesian University of Technology, 44-100 Gliwice, Poland; 2 Department of Building Processes and Building Physics, Faculty of Civil Engineering, Silesian University of Technology, 44-100 Gliwice, Poland; 3 Civil and Environmental Engineering Department, California State University, Fullerton, CA 92834, USA; 4 Department of Structural Mechanics, Faculty of Civil Engineering, VSB-Technical University of Ostrava, 70800 Ostrava, Czech Republic

**Keywords:** diffusion coefficient, diffusion model, chloride migration, standard methods, chloride ions, Fick’s second law

## Abstract

This research paper is the result of observations made during tests according to various standards carried out on behalf of industry. The article presents diffusion coefficient values calculated according to the thermodynamic migration model for twenty different concrete mixes and some selected mixes of the codified approaches known as ASTM 1202, NT BUILD 443, NT BUILD 492, ASTM 1556. The method used here, according to the thermodynamic model of migration, allows determination of the value of the diffusion coefficient after short studies of the migration of chloride ions into concrete and was described in earlier works by one of the authors. Unfortunately, when using standard methods, the values of diffusion coefficients differ significantly from each other. In each concrete, diffusion tests were carried out in the conditions of long-term natural diffusion to verify the values determined by standard methods and according to the thermodynamic model of migration. The analysis conducted for this research paper reveals that the chloride permeability test method according to the standard ASTM C1202-97 has an almost 2.8-fold greater dispersion of the obtained results compared to the thermodynamic model of migration. It was observed that the standard NT BUILD 492 has a 3.8-fold dispersion of results compared to the method with the thermodynamic model of migration. The most time-consuming method is the standard method NT BUILD 443. The largest 3.5-fold dispersion of values concerning the reference value are observed in that method. Moreover, a method based on a thermodynamic migration model seems to be the best option of all analyzed methods. It is a quite quick, but laborious, method that should be tested for a larger number of concrete mixes. A great advantage of this method is that it is promising for a wide range of concrete mixtures, both plain concrete and concrete with various additives and admixtures, as well as high-performance concrete.

## 1. Introduction

The rate of chloride ion penetration into fully and partially saturated concrete is described by diffusion parameters. The process of chloride ion penetration into concrete is described by Fick’s diffusion equations and the Nernst–Planck equation in the migration process [1,2]. Therefore, a very important issue in the case of reinforced and prestressed concrete structures is the precise determination of the diffusion coefficient value. Currently, there are many valid standard methods for determining the value of the chloride diffusion or migration coefficient [3]. Firstly, the method contained in two standards, ASTM C1556 [4] and NT BUILD 443 [5], can be mentioned. However, it is a long-term method (the test lasts *t* = 5 weeks, diffusion alone) and is effective in case of ordinary Portland cement-based concretes, while in testing modern concretes with high resistance against chloride penetration, this time may be too short to obtain a reliable distribution of ion concentration over the thickness of the sample. Due to the long duration of some experimental investigation methods, some other methods use an electric field to accelerate the penetration of chloride ions into concrete. In the method according to the standard ASTM C1202 [6], the electric charge passing through a given sample is measured and, on this basis, the chloride permeability through the concrete is assessed. Chloride permeation testing using an electric field was first conducted by Whiting [7]. This method, however, is not accurate, since it measures the charge transferred not only by chloride ions but all ions that are present in the concrete pore liquid, including OH^–^ ions. The other disadvantages of this method include the fact that measurements are made before the steady state is reached in the migration process and that the applied high voltage leads to an increase in temperature [8]. The value of the diffusion coefficient can be computed from the measured charge by using the Nernst–Einstein equation [9]. According to this standard [6], an accelerated method of measuring electric charge can be used in the case of concrete, in which it was first considered to have a fairly good relationship between the results enclosed in the test and the test results established in a different standard AASHTO T259-02 [10]. However, the obtained result of the method contained in the AASHTO T259-02 standard [10] also contains some inaccuracies, because the method does not depend on pure diffusion of chlorides. The chloride penetration is affected also by the sorption effect because dry samples are prepared for testing. Another problem related to the standard test ASTM C1202 [6] is that any addition of a material with higher conductivity, e.g., dispersed reinforcement or calcium nitrite admixture inhibiting corrosion processes, may lead to erroneous values of the specific diffusion coefficient based on the measured charge flowing through the sample.

The method of testing chloride migration through concrete, described by Tang and Nilson [11,12,13], was adopted as the standard test NT BUILD 492 [14]. This method allows for determination of the chloride ion migration coefficient, which determines the degree of concrete resistance to the penetration of chloride ions but cannot be compared with the diffusion coefficient obtained based on diffusion methods. In addition, the colorimetric method, which is used to determine the depth of chloride penetration, may be a source of inaccuracy. The visually determined change in concrete color depends not only on the concentration of the indicator containing silver chloride but also on the amount of hydroxide ions in the concrete [9,15].

Each method has different test conditions (concentration of the chloride source solution, type of target solution, different test duration), and the results of diffusion or migration coefficients obtained with different standard tests differ from each other when testing one concrete [16]. Now arises the question of which of the methods and the value of which coefficients should be applied to numerically forecast concrete constructions’ durability. Due to the ease and speed of electric field accelerated tests, there has recently been a trend to use quick methods of measuring the flowing charge, concrete resistance, and conductivity and convert the obtained values into the value of the diffusion coefficient [17]. However, researchers tend to forget that such tests should earlier be confirmed by tests under diffusion conditions (which is recommended by standard ASTM C1202 [6]), especially when deciding to perform durability forecasting using the coefficients determined in this way [18,19,20].

Additionally, when determining the diffusion coefficient with one method in samples with different maturation times, it was observed that the value of the diffusion coefficient changed with time. Thus, the diffusion coefficient is not a constant value but one that varies in time. The change of the diffusion coefficient was determined by introducing an exponential dependence aging factor whose value is determined experimentally for a given concrete and was introduced by Tang [11,21,22] and Nillson [23], followed by Body et al. [19,24] and Stanish et al. [25]. Taking this into account has a large impact on the expected durability of the structure [26,27].

Changes in the diffusion coefficient are, on the one hand, related to the processes of hydration and concrete maturation, i.e., improving its porosity and protective properties over a longer period of use. On the other hand, the interaction of chloride ions with the cement matrix (C_3_A, C_4_AF) can lead to changes in concrete porosity due to the formation of corrosion products such as: Friedel’s salt, basic calcium chloride, and, in the case of reaction with monosulfate, ettringite. Chloride binding could decrease concrete porosity as a result of a filling effect. These processes, by binding free chloride ions, slow down the rate of chloride ion penetration into the concrete. However, when unfavorable conditions occur, such as carbonation of concrete, as a result of which there is a decrease in the pH of concrete, these products decompose, releasing bound chloride ions. These products, as a result of increasing their volume, may also contribute to the formation of microcracks and damage to the concrete structure [28].

Several comparative analyses of some selected code methods were also carried out [16,29]. In some publications comparing the methods, we can find a good relationship between the coefficients determined by them, especially when the comparison concerns only a small number of methods and a small number of tested concretes [30,31]. For instance, in [32], the authors compared electrochemical methods for determining the diffusion coefficient. The values of the diffusion coefficients obtained based on the determined charge according to the ASTM C1202-91 [6] methods and the directly measured conductivity in the AASTHTO T259-02 [33] test were compared. A good relationship was obtained between the determined coefficients; however, the determined values were not compared with the actual distribution of chloride ion concentration in the concrete or with the method of determining the coefficient based on long-term diffusion methods. In reference [34], the authors determined the value of the diffusion coefficient using accelerated methods: rapid chloride permeability test (RCPT) according to ASTM C 1202 [6] in concrete after 28 and 91 days of maturation; rapid migration test (RMT) according to NT BUILD 492 [14] in concrete after 28 and 91 days of maturation; and bulk diffusion test (BDT) according to NT BUILD 443 [5] in concrete after 28 days of maturation using chloride ion immersion for 150 days. The average coefficient of variation (CV) of the mixtures during the tests is 21%. For means of comparison, this value is close to that accepted (20.2%) in the ASTM C1556 [4], the American standard equivalent to the NT BUILD 443 [5] for tests performed at different laboratories. A higher correlation can be identified between the test results of NT BUILD 443 [5] with NT BUILD 492 [14], presenting a CV between both test results of 13%, similar to the CV accepted (12.3%) by the ASTM C 1202 [6] standard.

In reference [35], the values of chloride diffusion coefficients in the concrete of different compositions were determined based on migration tests carried out for 600 hours on samples with a thickness of 50 mm using a voltage of 12 V and a source solution of 0.6 M NaCl. Then, as in the standard NT BUILD 492 [14], when determining the depth of chloride penetration, the DT value was determined with a transient flow of the chloride stream. The value of the DNP coefficients was also determined according to the Nernst–Planck equation after determining the chloride flow and D_σ_ based on a direct measurement of concrete conductivity. The exact values of coefficients were not given, but the following relationship was observed for all concretes: D_T_ > D_σ_ > D_NP_, where the values of the coefficient determined in the transient state were ten-times higher than the values determined in the steady-state flow and several times higher than the values determined from the conductivity measurements. Furthermore, Tang stated in his works [36,37] that the effective diffusion coefficient determined from diffusion or migration tests is not a constant value but a complicated function depending on changes in chloride concentration. On the other hand, in [38], the relationship between the content of chlorides penetrating the concrete during the migration test according to ASTM C1202-91 [6] and the depth of chloride ion penetration determined by the colorimetric method was analyzed, and it was found that the color change of concrete in the colorimetric test of the depth of chloride penetration occurs, on average, at concentration values from 1.13 to 1.14% of the chloride weight concerning the cement weight. However, in [39], this value ranged from 0.36 to 0.8% of the cement mass as the concentration of total chlorides.

In reference [40], a nonlinear relationship was noticed between the coefficient determined by the method according to ASTM C1556 [4] and the value of the charge determined by the method according to ASTM C1202-91 [6]. A relationship was proposed, thanks to which it is possible to estimate the value of the diffusion coefficient based on the determined charge, with the proviso that it is valid only for 56 concretes with the same compositions as those considered in this publication.

The authors of reference [41] analyzed the data contained in the literature on the determination of the diffusion coefficient using methods according to the standards NT BUILD 492 [14] or NT BUILD 443 [5] in 160 concrete mixes with an age of 28 days to 182 days. There were also other variables included, such as w/cm ratio from 0.30 to 0.70; different types of SCM, namely, silica fume, fly ash, ground granular blast furnace slag, and limestone with various degrees of replacement; aggregate volume fraction; cement content; and maximum aggregate size. These data were used for the critical evaluation of the accuracy and precision of the proposed analytical models for quantifying the diffusion coefficient of concrete chlorides. The introduction of so many models aimed at simplifying the procedure of determining the diffusion coefficient introduces even more confusion and differentiation of the obtained values, and it is still not known which of them will be appropriate to adopt when predicting the durability of reinforced concrete structures.

In reference [42], a completely different issue related to the penetration of chloride ions into concrete was considered. The influence of the location of the structure and the prevailing climate on the concentration of chloride ions in the outer layer of concrete with a thickness of 10 mm was checked here. This value obviously has a large impact on the rate of penetration of ions into the concrete; however, the value of the estimated diffusion coefficient is also of great importance, as it is a parameter determining the rate of penetration of these ions in water-saturated concrete. However, in reference [43], the value of the diffusion coefficient was determined using one standard method: accelerated migration testing according to the NT BUILD 492 [14] standard. The influence of various concrete additives affected the change of the concrete microstructure and its relationship with the obtained value of the determined diffusion coefficient. However, in this work, the standardized methods for determining the diffusion coefficient were analyzed and compared with a relatively new method according to the thermodynamic model.

In reference [44], two methods of diffusion coefficient determination were compared, and attention was paid to the impact of the time of testing and the duration of the test itself. A simple and fast method according to the standard [45], where the value of the diffusion coefficient at different times of sample maturity was determined, was compared with the standard method NT BUILD 443 [5]. Differences in the obtained values were attributed to the age of the tested concrete, and attempts were made to eliminate them with the aging coefficient. However, the comparison was carried out only for one concrete.

On the other hand, in reference [46], the focus was on the changes in the properties of the micro- and macrostructure of the concrete itself during the action of an aggressive environment containing groundwater that contained NaCl-MgSO_4_. In early erosion, as the of MgSO_4_ concentration increased, corrosion products were deposited in pores and cracks, which sealed the pore structure and reduced ion diffusion rates, thus inducing deterioration of sample macroscopic properties.

Interesting research was presented in [47]; it was stated in this work that there is significant difference between the results of the diffusion and migration coefficient (which is up to eight-times lower). When different testing procedures are used, different chloride penetration resistance is measured for the same concrete. It was also demonstrated that the rapid chloride migration test (RCPT2) is a sensible method of evaluating the chloride penetration resistance of concretes made of different types of cements. However, this test may be used only to a limited extent to compare protective properties of concretes against chloride penetration. It cannot be used to estimate the service life of steel-reinforced concretes because it does not specify the time after which the chloride ion content achieves the threshold level on the reinforcement surface.

It is possible to determine the chloride flow equation and formulate the inverse task of this equation based on the thermodynamic model. This approach to the problem allows for theoretically justified averaging of the experimentally obtained results. This approach is described in detail in reference [48], and examples of the method’s application for a wide range of concrete mixtures are described as well in [49,50,51,52].

This paper is a result of observations made during the tests according to various standards conducted upon the request of industry. Moreover, the reference tests were carried out due to concerns about the effect of different concrete mixtures on the performance of standardized testing procedures. Therefore, the article presents the values of the coefficient calculated according to the reference method and the relatively newly proposed thermodynamic model [48] of chloride migration for twenty different concrete mixtures. Concrete mixtures of different compositions and manufacturing technologies were tested to determine the diffusion coefficient by the thermodynamic migration model method. As mentioned above, for some of the concrete mixtures, tests were carried out following the standards, as these concretes were tested at the request of concrete producers, and the ordering party ordered tests using standard methods. Selected standard methods were used to determine the diffusion coefficient: ASTM 1556 [4], ASTM 1220 [6], NT BUILD 443 [5], and NT BUILD 492 [14].

### Significance

The exact determination of chloride ions’ diffusion coefficient is of great importance to the process of designing reinforced concrete structures with set durability. There is a wide array of diffusion coefficient-determining methods. Unfortunately, there is no homogeneity between the values of diffusion coefficients obtained from the same mixture with the use of different methods. This fact makes it hard for designers and producers to adjust concrete mixes to the durability standards since it is difficult to access which particular specification should be applied when determining the diffusion coefficient. This research paper presents a series of diffusion coefficient values obtained with the use of various standard methods and a newly designed method based on a thermodynamic migration model. The values were compared to the reference method. The reference method is based on fitting the curve based on the smallest value of the mean square error to the results of the distribution of chloride ion concentrations in concrete obtained from longer diffusion tests.

Moreover, the aim of the article is to draw attention to fact that with the increasingly diverse concretes used today, the current codified methods may not provide consistent results with different concrete mixtures. Therefore, it is a challenge within the concrete industry to find a universal yet feasible method for the evaluation of resistance against chloride penetration that will work in all types of concrete.

## 2. Materials

The research was carried out with several series of concrete. In the first series, five ordinary concretes (C1–C5) with a similar composition were tested, differing in the type of cement used. In the second series, three ordinary concretes (C6–C8) with a slight difference in composition were tested. Additionally, the C8 concrete has a sealing admixture according to the manufacturer’s recommendations. Self-compacting concretes (SCC1–SCC4) were used in the third series. Self-compacting concretes (SCCS0–SCCs100) modified by different volumes of the granulated imperial smelting process (ISP) were used in the fourth series. The fifth series consists of concretes (CP1–CP2) used in prestressed structures. Concrete CP1, which is used for the production of HC-500 floor slabs, was tested. The experimental results for concrete CP1 were partially published in reference [45] comparing different methods of concrete testing; however, it was compared only to one type of concrete.

Concrete CP2, which is used for the production of concrete lintel beams, was tested. Concrete with a w/c ratio of 0.3, based on portland cement CEM I 42.5 R (260 kg/m^3^) and natural rounded aggregate at 0–2 mm (800 kg/m^3^), as well as gravel at 2–8 mm (800 kg/m^3^), was tested.

In the sixth series, HSC1 high-strength self-compacting concrete was tested. The mixture is designed to be self-compacting with a spread of 740 mm class SF2 based on testing according to the BN EN-206 [53] standard code. It should be mentioned that the experimental results for HSC1 concrete were published in part already in reference [48], the experimental results for concretes of the first series were partially published in references [49,50], and those for concretes of the fourth series were published in part already in references [52]. The most reliable value of the diffusion coefficient among the range of coefficients determined based on the thermodynamic model of migration, taking into account the nonstationarity of the process, was, in the aforementioned works, determined differently than in the present work. The detailed compositions of mixes including their mixture ID are presented in Table 1.

The selected material properties of the tested concrete were investigated namely as compressive strength, volumetric weight, and permeable porosity. The permeable porosity of each mature, hardened concrete was determined in samples with a volume of about 10 cm^3^ placed in a kiln at 60 °C until a constant weight was obtained. The sample was then saturated with water by immersion until it reached constant weight. The floating mass of the sample was then measured with a laboratory hydrostatic balance. Finally, the open porosity was calculated, as listed in Table 2. The detailed chemical composition of the used cements is given in Table 3.

From each type of concrete, apart from CP1 and CP2, samples of concrete made in the shape of a cylinder with a diameter of 100 mm and a height of 50 mm, were tested. The molds were directly cast to a height of 5 cm, and the direction of chloride ion penetration for all samples was the same from the top of the sample. In CP1 concrete, test specimens were cut from ready-made elements from HC-500 floor slabs [49] and, in CP2 concrete, from lintels using a diamond drilling ring. The cut samples had a diameter of 80 mm and a height of 50 mm. Samples were cut from ready-made elements one year after their production in the factory. The direction of chloride ion penetration from the top of the sample was used for each method. Three samples from every type of concrete were used in each method.

## 3. Experimental Investigation

The degree of maturity of concrete has a great influence on the rate of chloride ion penetration into the concrete. As a result of the hydrolysis and hydration of the cement minerals, the binder gradually transforms into a hardened cement paste. The process runs at a specific speed, depending on the fineness of the cement, the water/cement ratio, temperature, and chemical and mineral composition. After the standard time of 28 days, the structure of the grout is formed; however, it still changes even with a significant slowing down of the hydrolysis and hydration reactions. Therefore, tests of concrete susceptibility to penetration of substances from the outside can be performed after the “calmed down material”. The term “calmed down material” is understood as a slurry, mortar, or concrete after 3 months, when the processes of hydration and hydrolysis of cement minerals have reached a significant degree of advancement. The solid phase is then in a state close to equilibrium with the liquid phase.

Studied concrete mixtures were tested with the reference thermodynamic method and other methods based on the contractor’s choice. Table 4 summarizes the tests performed on studied mixtures. The respective methods are described below.

### 3.1. Permeability of Chloride Ions According to the American Standard ASTM C1202

For selected concretes, the chloride permeability test through concrete was carried out using the electric field adopted as a standard test in the standards AASHTO T 277 [2] and ASTM C1202-97 [6]. It is marked as RCPT1 herein. The electric charge passing through the given sample was measured, and on this basis, the chloride permeability through the concrete was assessed. The permeability of concrete was determined depending on the load Q flowing through the sample. The value of the flowing load was obtained for each of the three samples. Based on the determined charge, the value of the diffusion coefficient was also calculated using the Nernst–Einstein equation [9]:

(1) $$D_{NE}=\frac{RT}{z^{2}F^{2}}\frac{t_{i}}{C_{i}\gamma_{i}\rho_{BR}},\;\rho_{BR}=\frac{100}{\sigma },\sigma =\frac{QL}{VtA}\;\;$$

where $D_{NE}$ is diffusion coefficient (m^2^/s), R is universal gas constant (J/Kmol), T is absolute temperature (K), z is the valence of ions (-), F is the Faradays constant (C/moL), $t_{i}$ is 1 transport number of chloride ions (-), $\gamma_{i}$ is 1 activity coefficient of chloride ions (-), $C_{i}$ is the concentration of chloride ions (moL/m^3^), ${}_{BR}$ is volumetric resistivity (Ωm), σ is conductivity (Ωm^−1^), *L* is sample thickness (m), *V* is electrical potential (V), *A* is cross-sectional area of a sample (m^2^), and *t* is time (s).

The value of the measured load and the diffusion coefficient values calculated on their basis for selected concretes, mean values of these coefficients, and standard deviations are presented in Section 4.5.

### 3.2. Permeability of Chloride Ions According to the Norwegian NT Standard BUILD 492

#### 3.2.1. Test Method

Another method used was described in the Norwegian NT standard BUILD 492 [14]. It is marked as RCPT2 herein. Concrete discs with a thickness of 50 mm and a diameter of 100 mm were used for the tests. The samples were soaked with lime water under vacuum conditions similar to the ASTM C1202-97 [6] standard method. The duration of the test and the value of the applied voltage depend on the current intensity determined at the beginning and are computed according to the table of the standard NT BUILD 492 [14]. There was no need to tilt the samples because the chloride ion source solution was in the upper reservoir, similar to the research proposed by Andrade [54], which allows for the free volatilization of hydrogen ions formed on the cathode. At the same time, three samples made of a given concrete were tested.

#### 3.2.2. Determination of the $D_{T}$ Migration Coefficient

After the test, one of the three tested discs was divided into two parts along its axis and then sprayed with a 0.1 M solution of silver nitrate AgNO_3_, and after about 15 min, the depth of chloride penetration $x_{d}$ was measured using the colorimetric method (based on a visually determined color change at the edge of the concrete sample). The area containing high chloride concentration turns silvery white due to the formation of AgCl derived from the reaction between Ag^+^ and Cl^−^. The area containing low concentration of chloride or no chloride turns into brown due to the formation of Ag_2_O from the reactions between Ag^+^ and OH^−^. Following the NT BUILD 492 [14] standard, the $D_{T}\;$ migration coefficient was calculated according to the formula:

(2) $$\alpha =2\sqrt{\frac{RTL}{zFU}}erf^{-1}\left(1-\frac{2c_{d}}{c_{0}}\right),\;\;\;\;\;\;\;\;\;\;\;D_{T}=\frac{RTL}{zFU}\frac{x_{d}-\alpha \sqrt{x_{d}}}{t_{d}}$$

where $c_{0}$ is the concentration of chlorides in the source chamber and the concrete, respectively, to the depth $x_{d}$; $t_{d}$ is test duration (hours); L is element thickness, (mm); U is the value of the applied voltage; $c_{d}$ is chloride concentration at which the color changes (0.07 M for OPC concrete); $c_{0}$ is chloride concentration in the cathode solution (2 M); $erf^{-1}\;$is the inverse of the error function.

The maximum value to which chloride ions penetrated was observed using a 1 mm graduated scale and was taken as the *x_d_* value from which the migration coefficient was calculated according to the formula given in this standard. It can therefore be assumed that the error of measurement is 0.001 m, and therefore, the error of the coefficient determined is 0.06 × 10^−12^. A large discrepancy can be observed between the values of the chloride penetration depth read based on the color change of the tested concretes and the actual chloride concentrations determined at a given depth as presented in Section 4. These differences may result from the different chemical compositions of the cement used and the different porosity of the tested concretes, much like in the references [15,39]. Alkalinity of concrete, concentration and volume of sprayed AgNO_3_ solution, and volume of pore solution have effects on measured chloride concentration at the color change boundary. An incorrectly read value of the chloride penetration depth using the colorimetric method may lead to large errors when determining the value of the diffusion coefficient according to NT BUILD 492 [14]. The concentration of hydroxyl ions (or pH value) in a concrete pore solution has a great influence on the critical chloride concentration at the color change boundary [38].

All tested profiles were presented in data set [55]. The $D_{T}\;$migration coefficient values calculated based on Equation (2) for selected concrete mixtures are presented in in Section 4.5. The migration coefficient value was calculated according to Equation (2), as mentioned above, based on the colorimetric method (with 0.1 M AgNO_3_), and silver nitrate solution determined *x_d_* depth of chloride ion penetration, as mentioned above.

#### 3.2.3. Determination of the $D_{migr}^{1}$ Migration Coefficient

Then, in the two samples remaining after the test described in point Section 3.2.1, the distribution of chloride ion concentration was determined directly in the tested concrete. For this purpose, layered concrete grinding was carried out using a specialist Profile Grinding Kit from Germann Instruments, and the distribution of chloride ion concentration was determined using the method described in point (Section 3.6).

The applied test, marked as NSC1, is based on the procedure for the diffusion coefficient proposed in [56]. Computation of the diffusion coefficient was conducted after performing chloride ion penetration enhanced by an electrical field according to NT BUILD 492, where the solution of the Nernst–Planck equation, after assuming some simplifications, was obtained in a form similar to the expression of pure diffusion, taking into account a certain multiplier expressing the influence of the electric field. The value $\;D_{migr}^{1}$ of the migration coefficient were determined by comparing the calculated distribution of chloride ion concentration according to the known solution of the diffusion equation with the concentrations of these ions, determined during the concrete migration tests.

(3) $$C_{cal}^{1}\left(x,t\right)=C_{0,cal}^{1}\left(1-erf\frac{x}{2\sqrt{D_{migr}^{1}t}}\right),\;D_{dif}^{1}=\frac{hRT}{FzU}D_{mig}^{1}$$

where $C_{0,cal}^{1}$ is calculated chloride ion concentration on the edge of the element, (%) is chloride mass to cement mass, $C_{cal}^{1}\left(x,t\right)\;\mathrm{is}\;\mathrm{the}\;$concentration of chloride ions calculated according to the Equation (3) at a distance x from the edge of the element, erf is Gaussian error function, and *t* is time (s).

The $D_{migr}^{1}$ migration coefficient values according to the Equation (3) for selected concretes are presented in Section 4.5.

#### 3.2.4. Determination of the $D_{dif}^{1}$ Diffusion Coefficient

In addition, in the last stage, the value of the diffusion coefficient was determined using the relationship between the diffusion and migration coefficient proposed by Andrade [54] applied herein and marked as NSC2. It is based on a simplified relationship between the diffusion $D_{dif}^{1}$ and migration $D_{migr}^{1};\;$the coefficient was described in Equation (3). The $D_{dif}^{1}$ diffusion coefficient values according to Equation (3) for selected concretes are presented in Section 4.5.

### 3.3. Permeability of Chloride Ions According to Norwegian Standard NT BUILD 443 and American Standard ASTM 1556

The test, marked as CPT, was carried out following the standards NT BUILD 443 [5] and ASTM C 1556-03 [4]. Concrete discs with a thickness of 50 mm and a diameter of 100 mm were used for the tests. All surfaces of the samples were insulated with resin, except for the top surface. Then, the samples were placed in lime water until completely saturated and then stored in a closed container immersed in 16.5% NaCl solution for 5 weeks (35 days). The concentration level of chloride ions in the water extract obtained from the fragmented concrete collected in layers was determined using the Profile Grinding Kit device with a diamond drill and an attachment enabling the collection of concrete layers with a thickness of 2 mm to a depth of 20 mm. The instruments were used in accordance with the method described in Section 3.6.

The value of the *D* coefficient according to the methods included in the Norwegian standard NT BUILD 443 [5] and the American ASTM 1556 [4] is determined by adjusting the chloride concentration graph. This graph is obtained by calculating the distribution of chloride ion concentration expressed to the cement mass according to the solution of the diffusion equation with the concentrations of these ions determined in the test:

(4) $$C_{cal}^{1}\left(x,t\right)=C_{0,cal}^{1}\left(1-erf\frac{x}{2\sqrt{Dt}}\right)$$

To determine the best fit calculation of the diffusion coefficient complying with the results of the experiment, the mean squared error is calculated based on the formula:

(5) $$s=\sqrt{\frac{\sum_{i=1}^{n}{\left[c_{cal}\left(x,t\right)-c\left(x,t\right)\right]}^{2}}{n-1}}$$

where *c*(*x*,*t*) is chloride ion concentration that was measured during the test at a distance *x* (mm) from the edge of the element (%), *t* is time (s), *n* is the number of concrete layers in which the chloride concentration was determined. The determined values of the diffusion coefficient are presented in Section 4.5.

### 3.4. Diffusion Test of Chloride Ions in Concrete

**Figure 1:**
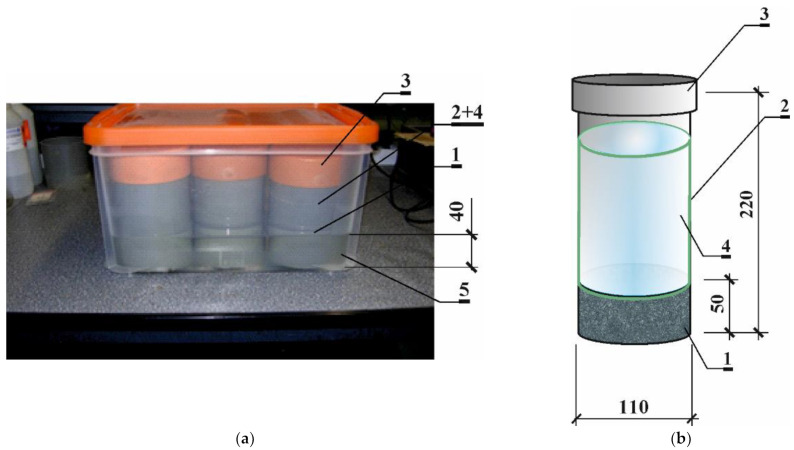
View of the stand (**a**) and the research elements (**b**) in the diffusion test: 1—concrete research element, 2—tank for source 3% NaCl solution, 3—conventional plugs to reduce evaporation, 4—3% NaCl solution, 5—distilled H_2_O.

A modified method based on tests that were conducted according to both NT BUILD 443 [5] and ASTM 1556 [4] standards were applied and marked as TD1 (for *t*_1_) and TD2 (for *t*_2_). However, a modified test stand (Figure 1) and individually selected test duration for each concrete were used. The detailed diffusion test durations of all of the series are presented in Table 5.

Three samples of each type of concrete were tested after 3 months of maturation. The samples were placed in plastic sealed containers filled with water to a height of 50 mm. Test specimen 1, after coating with epoxy resin, was placed tightly in PVC pipes with a diameter of 110 mm, which, at the same time, were tanks for two source solutions of chlorides. These tanks were filled with a 3% NaCl solution to a height of 15 cm. The surfaces between the element and the pipe were additionally sealed with silicone, and the contact edges were sealed with acrylic mass. Tubes were covered with three conventional plugs to reduce evaporation, as shown in Figure 1.

After the penetration and the evaluation of the chloride profile, the chloride ion concentration distribution was determined using the method described in Section 3.6. It is also important to mention that there is a difference in the application of Equations (4) and (5). Instead of concentration expressed concerning the weight of cement contained in concrete, the density *ρ*^1^ of chloride ion mass calculated according to Equation (7) in Section 3.6 was included.

### 3.5. Determining the Value of the Diffusion Coefficient Based on the Thermodynamic Model of Migration

**Figure 2:**
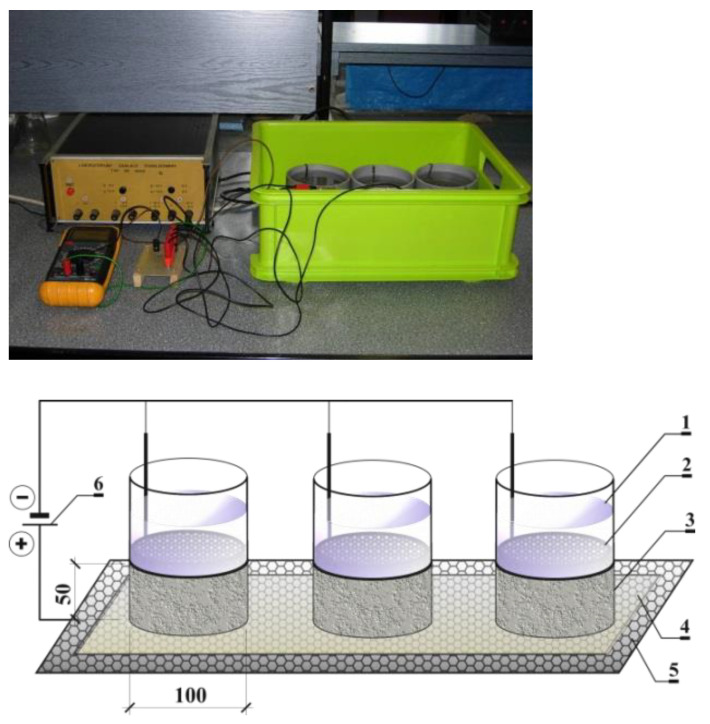
View and diagram of the test stand used in the experiment carried out according to the thermodynamic model of migration [42]: 1—plastic tanks filled with 3% NaCl solution, 2—cathode made of stainless steel, 3—concrete element, 4—damp sponge, 5—platinum-coated titanium mesh anode.

Modified tests, marked herein as TDX, were carried out on the test stand shown in Figure 2. Plastic tanks filled with 3% NaCl solution were tightly attached to the upper surface of the cylindrical samples with sides protected with epoxy resin. In tank (1), there is a cathode (2) made of stainless steel and adjusted in size to the cross-section of the tested element (3). The elements (3) were placed on a damp sponge (4), under which platinum-coated titanium mesh anode (5), immersed in water, was placed. Then, the samples were subjected to an electric field U = 18 V, inducing the migration of chloride ions. The test was conducted in two time intervals: *t*_1_ = 24 h and *t*_2_ = 48 h. NaCl solution was replaced every 24 h. During the whole period of tests, the temperature of the solution was constant at about 20 °C. After the chloride migration tests were completed, the source solution tanks were disassembled, and the elements were dried for 72 h under laboratory conditions. The layered grinding of concrete was carried out with a specialized device called the Profile Grinding Kit by Germann Instruments using the method described in Section 3.6.

The thermodynamic model of chloride penetration into the concrete ion flows in the pore liquid, marked as TDX, was analyzed by thinking out any representative volume element X. This element contains the concrete matrix, pores, and the aqueous solution. In the model, the matrix and water particles (solvent) are assumed to be the inert component α = 0, which is not directly involved in the process. The anions Cl^−^ and OH^−^ and cations Na^+^, K^+^, and Ca^2+^ are the components involved in this process. The concrete specimen was exposed to the electric field and chloride ions, which migrate in the aqueous solution. The electrodes connected to the source of direct current and applied to the specimen of height *h* cause the one-way migration of ionic components in the pore solution under the voltage U. After transformations, the diffusion coefficient of chloride ions *D*^1^ = 1/*Q*, which is the reverse of the diffusion resistance of the entire tested concrete zone with the range [44].

(6) $$D^{1}=\frac{\bar{j^{1}}\left(a\right)a\Delta t}{\frac{z^{1}FUg}{RTh}\left[\bar{\rho_{1}^{1}}+\bar{\rho_{2}^{1}}+\ldots +\bar{\rho_{n}^{1}}\right]\Delta t-B},\;B≅\omega \frac{z^{1}FUg}{RTh}\left(\bar{\rho_{1}^{1}}+\bar{\rho_{2}^{1}}+\ldots +\bar{\rho_{n}^{1}}\right)\Delta t.$$

In this expression, $\bar{j^{1}}\left(a\right)$ is the value of the mass flow of chloride ions passing through the plane situated at “a” distance x=a; $\bar{\rho_{1}^{1}}$, $\bar{\rho_{2}^{1}}$, and $\bar{\rho_{n}^{1}}$ are the averaged mass densities of ion Cl^−^ at midpoints of consecutive intervals $\left(0,g\right)$, $\left(g,2g\right)$, …,$\left[\left(n-1\right)g,a\right]$ in time Δt. The first component of the denominator defines the stationary part of the chloride ion flows, while the second component B defines the nonstationary part (in this paper, the value *B* = 0). In this expression, *z*^1^ is the ion valence, *R* = 8.317 J/moL·K is the universal gas constant, *F* = 96 487 C/moL is the Faraday constant, *U* is the voltage between the electrodes, and *h* is the specimen height.

According to the relations (6), the contribution of the nonstationary influence was estimated proportionally to the component expressing the stationary part. By inserting the proportionality factor with a range of ω = 0.1–0.5 into the equation, the value of the diffusion coefficient of chloride ions was estimated, taking into account the nonstationary course of the migration. For this paper’s purpose, the value of B = 0 and the value of the diffusion coefficient without the influence of process nonstationarity were selected. Based on the mass density of chloride ions determined on the thickness of the concrete cover at two different test times for the migration of chloride ions in the concrete, the values of the diffusion coefficient were determined according to Equation (6). The method is described in detail in reference [48]. The determined values of the diffusion coefficient for all concretes are presented in Section 4.5.

### 3.6. Determining of Chloride Ion Concentration Distribution in Concrete

**Figure 3:**
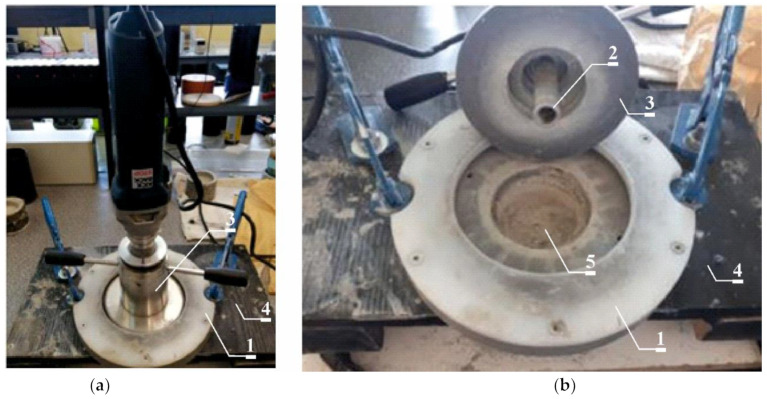
General view of the concrete grinding station (**a**), view of the samples from which the concrete material was ground (**b**). 1—grinding support plate, 2—high-performance grinding diamond bit, 3—handle cover with flange and counter nut, 4—station serves to hold the concrete sample, 5—concrete sample while grinding its surface.

Each time after carrying out the tests described in points Section 3.2, Section 3.3, Section 3.4 and Section 3.5, after drying the samples in laboratory conditions (72 h), the concrete powder was collected in layers. The ground concrete was collected using the “Profile Grinding Kit” from Germann Instruments (Evanston, IL, USA) as shown in Figure 3.

The diameter of the abraded surface is 73 mm and is limited due to the use of a special grinding support plate (1). The material is collected by abrasion using a grinding machine with a high-performance grinding diamond bit (18 mm in diameter) (2). The set for collecting concrete layers consists of a grinding machine placed in a special handle cover with flange and counter nut (3), which enables precise adjustment of the abrasion depth to the value of 2.0 mm. The station (4) serves to hold the concrete sample (5) while grinding its surface. The view of the station is shown in Figure 3.

In order to obtain sufficient mass of material necessary for chemical tests and in order to obtain average properties of the tested material, crushed concrete was combined from appropriate layers of a set of three samples tested under the same conditions. Water extracts were made from fragmented concrete, representing the averaged properties of individual layers of tested elements.

The values of chloride ion concentration c1 in model solutions obtained based on chemical analyses were assigned to the interval location of concrete taken from samples. The tests were carried out with the CX-701 multimeter of the brand “Elmetron” using an ion-selective electrode for determining the concentration of chloride ions. The mass density $\rho^{1}$ in the concrete of the samples was determined based on the test results for the concentration of c1 chloride ions in the model solutions. As the fragmented concrete was washed twice with water in a 1:1$\left(m_{ml}=m_{c}\right)$, so the volume $V_{ml}$ of the model liquid was estimated based on the volumetric weight of water $\gamma_{w}=1$ (kg/dm^3^). Taking into account that during the grinding of concrete, part of the concrete dust is lost, the volume $V_{c}$ of concrete (m^3^) was determined based on its volume weight $\gamma_{c}$.

(7) $$V_{ml}=\frac{m_{ml}}{\gamma_{w}}=\frac{2m_{c}}{\gamma_{w}},\;m^{Cl}=\frac{2c^{Cl}m_{c}}{\gamma_{w}},\;V_{c}=\frac{m_{c}}{\gamma_{c}}$$

The mass density $\rho^{1}$ of chloride ions (kg/m^3^), percentage of chloride content to the cement mass $\;C^{1}$, referring to the $\rho_{cem}$, and the constant mass density of cement in concrete (kg/m^3^) [48] was determined:

(8) $$\rho^{1}=\frac{m^{1}}{V_{c}}=\frac{2c^{1}\gamma_{c}}{\gamma_{w}},\;C^{1}=\frac{\rho^{1}}{\rho_{cem}}$$

$\mathrm{where}\;c^{1}$ is chloride ion concentration determined in the washed solution (kg/dm^3^), *m*^1^ is the mass of chloride ions in the volume, *V_ml_* is the mass of the of the model liquid (g), *V*_c_ is concrete volume (m^3^), $\gamma_{c}$ is concrete volumetric weight (kg/m^3^), and $\gamma_{w}$ is 1 (kg/dm^3^) in terms of water volume weight.

### 3.7. Advanced Method for Chloride Ion Concentration Distribution Detection

It is worth mentioning that there are advanced methods that allow detection of chloride content in a complete section of concrete samples, such as the X-ray fluorescence technique (XRF, see, e.g., [57]) and laser-induced breakdown spectroscopy (LIBS, see, e.g., [58]). However, advanced methods of determining the concentration of chloride ions and their analysis are not the subject of this work.

## 4. Results

### 4.1. Results of the Tests Carried Out According to the ASTM C1202-97 (RCPT1)

The “Proove’it” of brand “Germann Instruments” was used in the six-hour test under the electrical potential of 60 Volts. The resulting passed charges that indicate the resistance to chlorine penetration for the concretes C6, C7, C8, SCC1, SCC2, SCC3, SCC4, CP1, and HSCC1 are given in Table 6, as well as the respective diffusion coefficient computed according to Equation (1). Diffusion coefficients were obtained by measuring the charge in every sample of each concrete. Next, the mean value of coefficients and standard deviation σ were obtained. Based on the charge value and under the standard ASTM C1202-97 [2], the concrete C6 can be considered as having low permeability, and concretes C7, C8, SCC2, SCC3, and CP1 can be considered as having very low permeability. SCC1 and SCC4 concrete can be classified as medium-permeable concrete, and HSCC1 concrete is low-permeable concrete.

As can be seen in Table 6, the lowest resistance to the value of the transferred load was shown by concretes made of CEMI ordinary portland cement, with the exception of HSCC1 concrete, which contains silica fume, thanks to which the concrete obtained very low porosity and is characterized by very high tightness. Additionally, the use of basalt aggregate improves the resistance of concrete against the flow of electric current. Self-compacting concretes showed lower electrical resistance compared to ordinary concretes, interestingly, despite the higher content of cement. However, the mere change of the type of cement in the composition from portland to multicomponent resulted in an improvement in the concrete resistance in both ordinary and self-compacting concretes.

However, this information is very general; therefore, based on the determined electric charge, the value of the diffusion coefficient was also calculated using the Nernst–Einstein Equation (1). Since the calculations are based on the values of the measured charge, it is not clear if this charge is transferred by chloride ions only or by hydroxide ions; hence, the obtained values of diffusion coefficients do not determine the rate of chloride ion penetration.

Additionally, in reference [56], there were some discrepancies between the measured electric charge based on the ASTM C 1202-97 standard [2] and the amount of chloride penetrating the sample determined on the basis of diffusion tests, which is explained by the possibility of discrepancies in the assessment of the degree of permeability of concrete with the addition of fly ash and blast furnace slag granulates or other admixtures reducing the water content in concrete. On the other hand, the addition of various admixtures to concrete, e.g., those containing Ca(NO_3_)_2_, may increase the conductivity of the pore liquid due to the addition of ions from the admixture.

### 4.2. Results of the Tests Carried Out According to the NT BUILD 492 (RCPT2)

**Figure 4:**
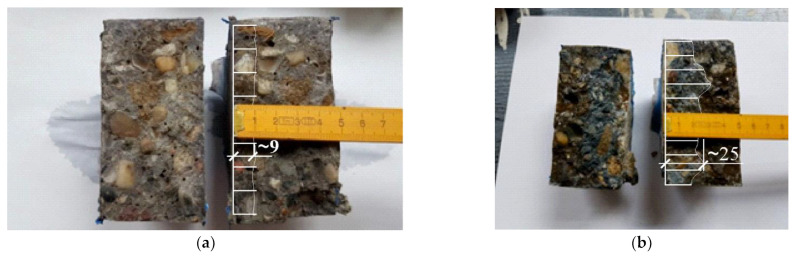
Chloride penetration depth *x_d_* = 9 mm-C6 concrete (**a**); *x_d_* = 25 mm-C7 concrete determined by the colorimetric method (**b**).

The chloride migration test was carried out for 96 h at the applied voltage of *U* ≈ 60 V. After the test, the discs were split into two parts along their axis, then sprayed with 0.1 M AgNO_3_ silver nitrate solution, and after about 15 min, the depth of the chloride penetration x_d_ was measured using the colorimetric method (based on the visually determined color change at the edge of the concrete sample). Figure 4 presents the sample result for concrete C6 and C7. A complete set of results is presented in the data set [55].

**Figure 5:**
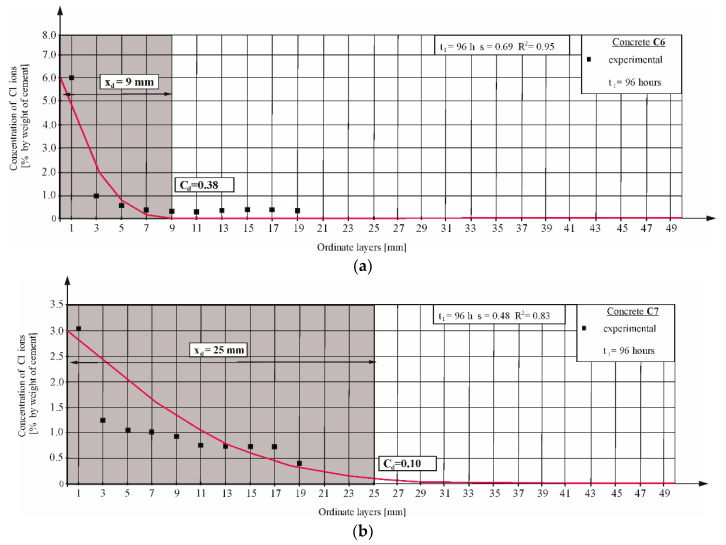
Chloride ion profile determined in migration tests according to NT BUILD 492 [14] and calculated using the Equation (5) in concretes: (**a**) C6; (**b**) C7.

The concentration level of chloride ions in the water extract obtained from concrete collected with layers was also determined as described in Section 3.6, which can be seen for the concrete C6 and C7 in Figure 5. For a complete set of results, refer to the data set in reference [55].

From the diagram of chloride concentration obtained in the migration tests lasting 96 h, it can be roughly predicted that for C6 concrete, the chloride penetration depth $x_{d}$ = 9 mm would correspond to the chloride concentration expressed per cement mass of approximately $c_{d}$ = 0.38% (Figure 5a). For C7 concrete, the chloride penetration depth $x_{d}$ = 25 mm would correspond to the chloride concentration $c_{d}$ = 0.1% (Figure 5b).

Similar inconsistencies were observed also in the case of other concrete mixtures, as can be seen in Table 7.

A large discrepancy can be observed between the values of the chloride penetration depth read based on the color change of the tested concretes and the actual chloride concentrations determined at a given depth. Therefore, all tested profiles were presented in the article. These differences may result from the different chemical compositions of the cement used and the different porosity of the tested concretes, much like in references [15,40]. An incorrectly observed value of the chloride penetration depth using the colorimetric method may lead to large errors when determining the value of the diffusion coefficient according to NT BUILD 492 [14]. The concentration of hydroxyl ions (or pH value) in concrete pore solution has a great influence on the critical chloride concentration at the color change boundary [38]. The computed diffusion coefficients are summarized in Section 4.5.

### 4.3. Results of the Tests Carried Out According to the Standard NT BUILD 443 and the American Standard ASTM 1556 (CPT1)

**Figure 6:**
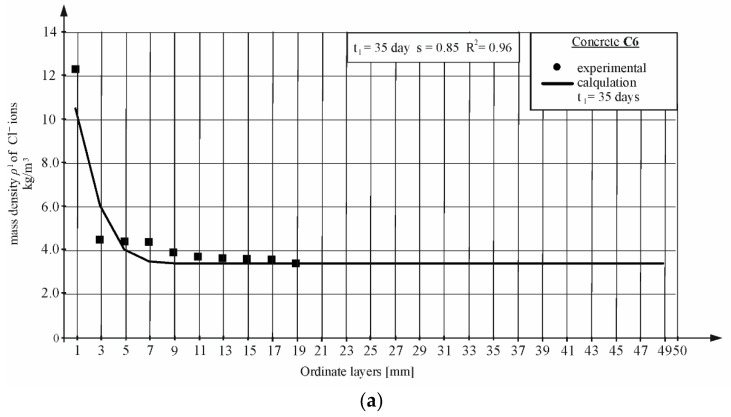

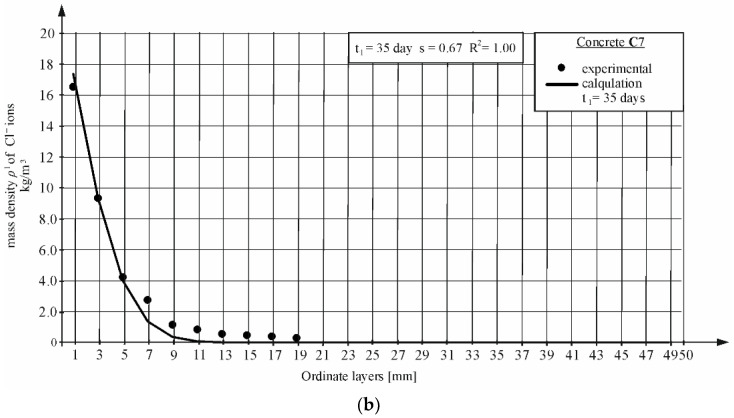
Chloride ion profile determined in migration tests according to NT BUILD 443 [5] and American ASTM 1556 [4] and calculated using the Equations (4) and (5). Concretes (CPT): (**a**) C6; (**b**) C7.

Figure 6 shows the results of diffusion tests carried out with the use of the method presented in the NT BUILD 443 [5] standard for chosen concretes C6 and C7. The tests were carried out at one time equal to *t*_1_ = 35 days with the use of source solution 16.5% NaCl. Then, by adjusting the obtained concentration curves to the values obtained from the tests, the values of the diffusion coefficient were determined based on the lowest mean square errors. Figure 6 shows examples of concentration curves calculated taking into account the values of the diffusion coefficients and the mass density of chloride ions obtained from the tests acquired in accordance with the equation. The graphs also show the *s* mean square error, based on which the data of the values of the coefficients and R^2^ coefficient of determination for the following curves were determined. The values of the diffusion coefficients for all concretes are given in Section 4.5 and the data set in reference [55].

### 4.4. Determining the Value of the Diffusion Coefficient Based on the Results of the Diffusion Test in Time *t*_1_ and *t*_2_ of Chloride Ions in All Concrete (TD1 and TD2)

**Figure 7:**
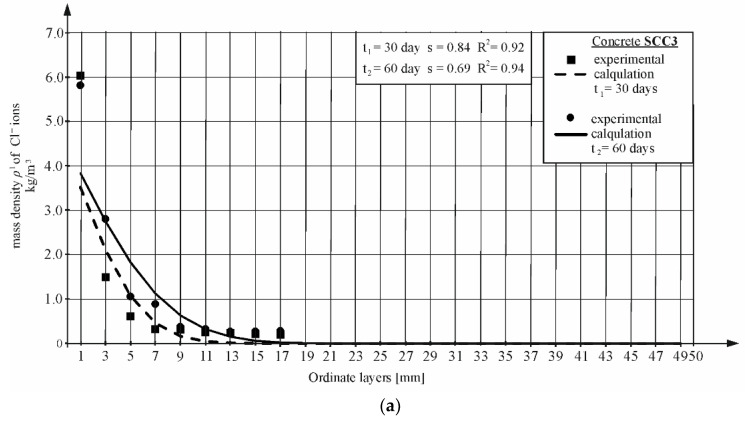

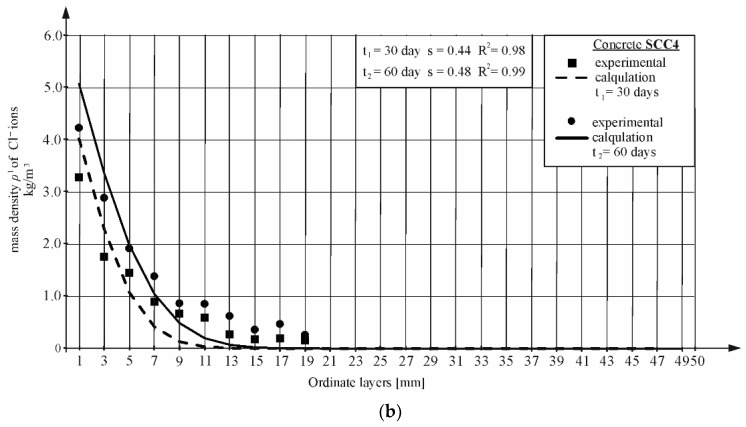
Chloride ion profile determined in diffusion tests in time *t*_1_ and *t*_2_ and calculated using the Equations (4) and (5) in concrete (TD1 and TD2): (**a**) SCC3; (**b**) SCC4.

**Figure 8:**
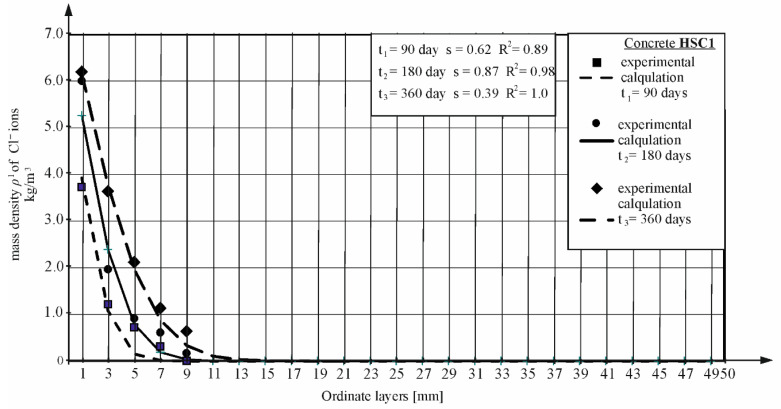
Chloride ion profiles were determined in diffusion tests in time *t*_1_, *t*_2_, and *t*_3_ and calculated using the Equations (4) and (5), HSC1 concrete (TD1 and TD2).

Most studies were conducted at two different times. Then, by adjusting the obtained concentration curves to the values obtained from the tests, the values of the diffusion coefficient were determined based on the lowest mean square error, similar to the method presented in both the NT BUILD 443 [5] and ASTM 1556 [4] standard and described in Section 3.3 of this article. Figure 7 and Figure 8 show the concentration curves for selected concretes, calculated by taking into account the values of the diffusion coefficients for all concretes given in Section 4.5. The graphs also show the *s* mean square error, based on which the data of the values of the coefficients and R^2^ of determination for the following curves were determined.

### 4.5. Comparison of the Value of the Diffusion Coefficient Obtained with Different Methods

Table 8 lists the values of the diffusion and migration coefficient determined by various methods. The table contains the values of the diffusion coefficient $\bar{D_{NE}}$ determined based on the charge measured during the migration test according to the standard ASTM C1202-97 [6] after applying the Nernst–Einstein Equation (1) (RCPT1). The next coefficient, $D_{T}$, as specified in the standard NT BUILD 492 [14], was computed based on migration tests, and the depth of chloride ion penetration was determined by the colorimetric method using Equation (2) (RCPT2). Successive values of the migration $\;D_{migr}^{1}\;$(NSC1) and diffusion coefficient $D_{dif}^{1}$ (NSC2) were determined using the method of fitting the concentration curve plotted according to Equation (2) to the results obtained in the migration test conducted according to the standard NT BUILD 492 [14]. Equation (2), which, in a simplified manner, considers a certain multiplier linking the diffusion flow with the migration of chloride ions, was used. The next diffusion coefficient *D* was determined based on diffusion tests carried out according to the standard NT BUILD 443 [5] and determined using Equation (4) based on the lowest value of the mean square error, Equation (5), between the values of the concentration of chloride ions determined computationally and experimentally (CPT). The next two values of the diffusion coefficient $D^{t1}$(TD1) and $D^{t2}$(TD2) were determined according to the method of fitting the concentration curve numerically determined following Equations (4) and (5) to the value of the chloride ion concentration distribution on the thickness of the concrete element obtained after diffusion tests in which the same test system was used for all concrete and the concentration of the source solution was the same for all tests. Another value of the diffusion coefficient D^1^ was determined based on migration tests, and Equation (6) was determined from the thermodynamic migration model (TDX).

Then, the analysis of the computed values was carried out by comparing, for each concrete, a percentage related to the value of the diffusion coefficient $D^{t2}$(DT2) determined by the curve fitting method after diffusion tests carried out at a longer time t2.

It is worth mentioning that detailed chloride profiles and chloride penetration depth are available as a data set [55].

**Figure 9:**
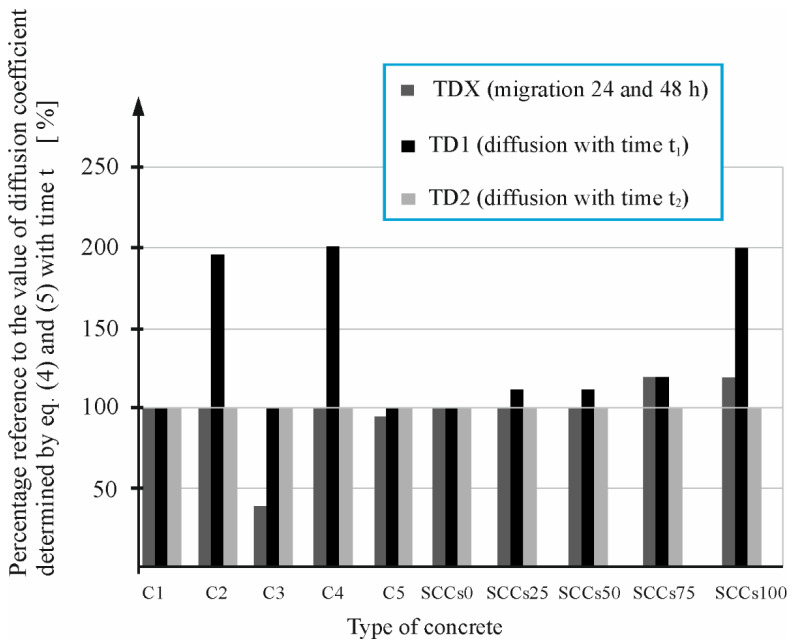
Percentage reference of diffusion coefficient (TDX) and values determined by Equations (4) and (5) at time *t*_1_ (TD1) and time *t*_2_ (TD2) to the reference value determined at time *t*_2_ (TD2): in C1–C5 concretes and SCCs0–SCCs100 concretes.

Figure 9 shows the percentage ratio of the diffusion coefficient $D^{1}$(TDX) determined after short migration tests based on the thermodynamic migration model [48]. This model concerns the diffusion coefficient $D^{t2}$(DT2) determined after a longer diffusion time using the curve fitting method NT BUILD 443 [5] for the concretes C1, C2, C3, C4, C5, SCCs0, SCCs25, SCCs50, SCCs75, and SCCs100.

**Figure 10:**
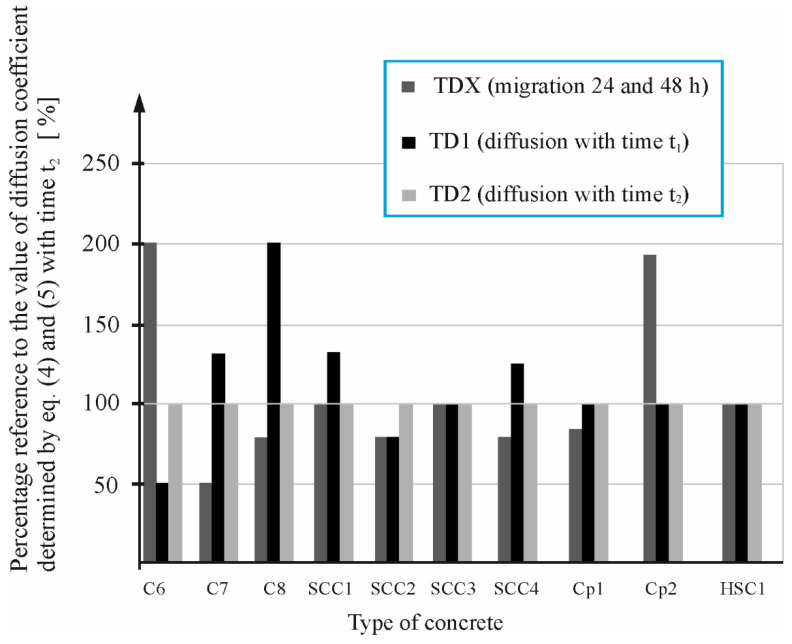
Percentage reference of diffusion coefficient (TDX) and values determined by Equations (4) and (5) at time *t*_1_ (TD1) and time *t*_2_ (TD2) to the reference value determined at time *t*_2_ (TD2): in C6–C8, SCC1–SCC4, Cp1, Cp2,HSC1 concretes.

Figure 10 shows the percentage ratio of the diffusion coefficient D^1^ (TDX) determined after short migration tests based on the thermodynamic migration model [48] in relation to the diffusion coefficient D^t2^ (TD2) determined after a longer diffusion time using the curve fitting method NT BUILD 443 [5] for the concretes: C6, C7, C8, SCC1, SCC2, SCC3, SCC4, CP1, CP2, and HSC1.

As observed from Figure 10, only in the case of the two concretes C6 and CP2, the values of the diffusion coefficient determined based on the thermos diffusion model [48] (TDX), the percentage ratio to the value of diffusion coefficient determined by Equations (4) and (5) with time *t*_2_ (TD2) is large and amounts to nearly 200%. On the other hand, observed results for concrete mixtures C3 and C7 were circa 50% that of the TD2. Similar performance was observed in the case of four concretes, C2, C4, SCCs100, and C8 (Figure 9 and Figure 10). The diffusion coefficient determined based on the curve fitting for diffusion tests after a shorter time *t*_1_ (TD1) also had a large percentage ratio to the value of diffusion coefficient determined with time *t*_2_ (TD2), which amounts to nearly 200%. In the case of nine concretes, C1, C2, C4, SCCs0, SCCs25, SCCs50, SCC1, SCC3, and HSC1, with the values of the diffusion coefficient determined based on the thermos diffusion model [48] (TDX), the percentage ratio equals 100%, whereas in the case of eight concretes, C1, C3, C5, SCCs0, SCC3, CP1, CP2, and HSC1, with the values of the diffusion coefficient determined based on the curve fitting for diffusion tests after a shorter time (*t*_1_) (TD1), the percentage ratio is equal 100%. To summarize, in Figure 9 and Figure 10, we can observe the average positional deviation from the base values, which are the values of the coefficients determined after longer tests. In the case of the coefficient determined from the thermodynamic migration model (TDX), it amounts to 21%, while in the case of the coefficient, the values determined after a shorter test (TD1) are 24%.

The large deviation of the coefficient obtained in the case of C2, C4, and SCCs100 concretes should be attributed to the instability of the process in the shorter test time. This instability may be caused by differences in the microstructure of the concrete samples and different chemical composition of the pore liquid. This diversity is due to the difference in the cement used in the case of C2 concrete (only in this, concrete CEM II/B-V 32.5 R cement was used) and in C4 concrete (here, CEM I/N/SR3/NA was used). On the other hand, the fact of replacing the total content of fine aggregate with slag in the SCCCs100 concrete could have caused a delay in the reactions of combining this slag with a smooth surface, which caused the process instability in the first phase of the experiment.

**Figure 11:**
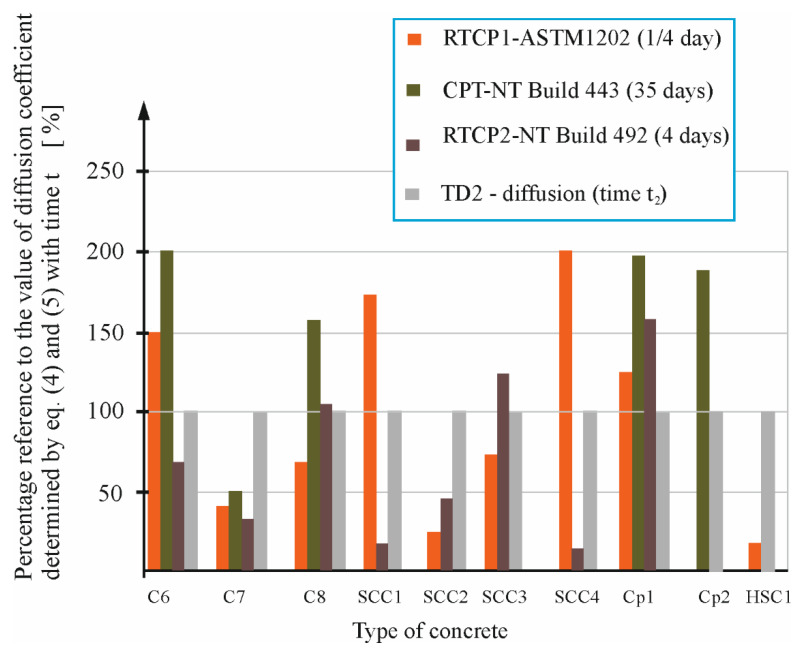
Percentage reference of the value of diffusion coefficient determined under the ASTM C1202-97 [6] (RCPT1), NT BUILD 492 [14] (RCPT2), and NT BUILD 443 [5] (CPT) standard to the value of diffusion coefficient (TD2) determined by Equations (4) and (5) with time *t*_2_.

Figure 11 shows the percentage ratio of the diffusion coefficients D_N-E_ (RCPT1), DT (RCPT2), and D (CPT) determined according to the ASTM C1202-97 [6], NT BUILD 492 [14], and NT BUILD 443 [5] standards, respectively, in relation to the diffusion coefficient D^t2^ (TD2), which was determined for the concretes C6, C7, C8, SCC1, SCC2, SCC3, SCC4, CP1, CP2, and HSC1 after a longer diffusion time using the curve fitting method.

As can be seen from Figure 11, among the coefficients determined by standard methods, we observed an even greater dispersion concerning the value of the coefficient determined from the thermodynamic migration model. In the case of the coefficient determined based on the ASTM C1202-97 [6] (RCPT1), the average positional deviation from the base values equals 59%, while in the case of standard method NT BUILD 492 [14] (RCPT2), this value is 71%, while in the case of standard methods NT BUILD 443 [5] (CPT), the deviation is 79%.

## 5. Summary

The evaluation of the performance of the procedures for the diffusion coefficient for chloride penetration was conducted on twenty different concrete mixtures. The study is accompanied by an evaluation of selected concretes according to codified methods (ASTM 1202, NT BUILD 443, NT BUILD 492, and ASTM 1556) and compared with the reference method and the proposed approach based on the thermodynamic migration model. The overall findings are summarized below:

- Of the methods considered, the most accurate method, while very time-consuming, is the natural penetration-based method for computing the diffusion coefficient, a curve fitting method for results obtained from a diffusion test lasting 60 or 180 days or longer (360 days) in the case of highly compact concretes (TD2). This method is the best representation of the natural diffusion process, and the diffusion coefficient values can be used in numerical methods modeling the phenomenon of chloride ion diffusion into concrete.
- The quickest and least labor-consuming method (RCPT1) used in the research is the chloride permeability test method according to the standard ASTM C1202-97.
  ○ However, it is an indirect method obtaining the value of the diffusion coefficient based on correlation with the charge passing through the concrete.
  ○ Although the method is faster, it can be used only for concretes for which the results are confirmed by using another long-term standard method.
  ○ In this method, there is an almost 2.8-fold greater dispersion of the obtained results compared to the thermodynamic model of migration [48]. A high standard deviation can be a result of the method drawbacks.
- The standard NT BUILD 492 [14] method (RCPT2) is also quite fast and requires relatively little effort.
  ○ The colorimetric method of determining the depth of chloride ion penetration may lead to large errors in concretes containing various types of cement, raising serious doubts.
  ○ There is also no clarity regarding the coefficient that was computed, as the standard specifies that it is a nondiffusion migration coefficient; therefore, it is not useful in the context of using the diffusion coefficient in models that allow prediction of the durability of reinforced concrete structures.
  ○ Additionally, a 3.8-fold dispersion of the results compared to the method with the thermodynamic model of migration was observed.
  ○ Standard deviation can be a result of using a method based on Equation (4), which was determined empirically by Tang and Nillson [11,12,13] based on chloride concentration distribution and in which $x_{d}$ was a depth at which chloride concentration $c_{d}$ obtains a constant value. However, the graph presented in Figure 11 shows that this value falls within the 0.012 to 0.55 scope, which is a result of colorimetric method imprecision.
- The most time-consuming and long-lasting method (CPT) is the standard method NT BUILD 443 [5].
  ○ Unfortunately, in this case, the largest 3.5-fold dispersion of values concerning the reference value are observed.
  ○ The reason for the dispersion may be a large difference in the concentration of used source solutions and the difference in the preparation of test samples that are saturated in lime water before the test.
- The next method (TDX) is utilized according to the thermodynamic migration model [48]. Thus, the method seems to be the best among the analyzed methods. It is a relatively fast yet labor-intensive method that needs more extensive testing with different concrete mixtures.
  ○ In this method, the smallest average positional deviation, which is 21% of base values, was also observed.
  ○ By using an electric field in the test method and solving a thermodynamic migration model [48], the value of the diffusion coefficient is obtained after short-term tests lasting usually 24 and 48 h or 48 and 72 h in the case of concretes with very compact structures.
  ○ It is based on two measurements that allows one to obtain the mean diffusion coefficient value and to include the effect of diffusion process nonstationarity related to the capacity of chloride ions to bind in the cement matrix [59].
  ○ It requires a greater amount of labor than other rapid migration methods (RCPT1 and RCTP2), but thanks to the use of an electric field, it is shorter than natural penetration-based approaches (CPT, TD1, and TD2).
  ○ It is a relatively fast testing procedure.
  ○ A great advantage of this method is that it is promising for wide range of concrete mixtures, both plain concrete and concrete with various additives and admixtures, as well as high-performance concrete, even though it was tested on a relatively low number of mixtures.
  ○ Even though there were three more significant differences from 20 mixture designs observed between the thermodynamic model and the base model, the overall match was better compared to the ASTM 1202, NT Build 493, or 492 standards.

## 6. Conclusions

It is important, both in the process of designing the concrete composition of new constructions and in assessing the protective properties of the concrete of existing structures, to correctly determine the value of the diffusion coefficient. One should be aware that the use of very easy and fast methods of determining the value of the diffusion coefficient from the measurement of the charge or the measurement of the depth of penetration of chloride ions may entail the possibility of committing gross errors. It is therefore a great challenge to find a method faster than the classic diffusion test but more universal and applicable to a wide range of concretes. Concretes are currently modified with various additives, and thus, the future effects of them are difficult to predict.

The material parameter describing the concrete resistance against chloride penetration was evaluated. The tests were performed according to various standards and complemented with reference thermodynamic models and natural diffusion tests.

It was observed that:

(1) The values obtained using standardized accelerated chloride penetration methods differed from each other, and it was difficult to find the relationship between them.
(2) The results of thermodynamic model matched with the model based on longer natural penetration better comparing to codified approaches.
(3) Moreover, the time needed for the thermodynamic test was in the order of days, making it of great importance in the preparation of an appropriate recipe for concrete used in the production of reinforced concrete structures.
(4) The presented results confirmed doubts about the application of standardized approaches for the fast evaluation of chloride diffusion coefficients.
(5) It is important to keep in mind its limited range of applications compared to traditional long-term penetration tests, adding therefore to the already mentioned concerns.
(6) Since each of the indirect methods might need calibration based on the material content, further research might be focused on the evaluation of the mixture design on the results of the indirect determination of the chloride migration coefficient.

Structural design taking into account the appropriate value of the diffusion coefficient will allow for longer durability and savings related to repairs and failures of such structures.

## Figures and Tables

**Table 1:** Composition of studied concrete mixtures all of series.

Series	Mixture ID.	Sand (0–2) * mm [kg/m^3^]	Gravel (2–8) * mm [kg/m^3^]	Gravel (8–16) * mm [kg/m^3^]	ISP Slag [kg/m^3^]	Cement [kg/m^3^]	Additives [kg/m^3^]	w/c
1	C1	722	512	2271	-	681	-	0.5
	C2							
	C3							
	C4							
	C5							
2	C6					368	1.84 ***	0.4
	C7							
	C8							
3	SCC1		-	2308		580	2 ***	0.3
	SCC2		-	2257		579	1 ***	0.3
	SCC3		-	2198		555	1.5 ***	0.3
	SCC4		-	2187		508	1 ***	0.4
4	SCCs0	750	570	300	0	450	13.5 *** 1.8 *****	0.4
	SCCs25	562.5			217.5			
	SCCs50	375			435			
	SCCs75	187.5			652.5			
	SCCs100	-			870			
5	CP1	580	671 **	633 **		550	6.2 ***	0.3
	CP2	800	800	-		260		0.3
6	HSC1	580	671 **	633 **	225 ****	480	7.49 ***	0.2

* grain diameter range, ** crushed basalt, *** plasticizer, **** silica fume, ****** stabilizer.

**Table 2:** Properties and compressive strength of studies concrete mixtures all of the series.

Mixture ID	Type of Cement	Compressive Strength [MPa]	Volume Weight [kg/m^3^]	Porosity [%]
C1	CEM I 42.5 R *	54.2	2271	12
C2	CEM II/B-V 32.5 R *	45.8	2241	10
C3	CEM III/A 42.5 N-LH/HSR/NA *	49.5	2269	7
C4	CEM I/ N/SR3/NA *	58.4	2258	9
C5	CEM IV/B (V) 32.5 R–LH/NA	46.5	2280	10
C6	CEM I 42.5 R	62.4	2309	9
C7	CEM III/A 42.5 N-LH/HSR/NA *	63.9	2273	9
C8	CEM III/A 42.5 N-LH/HSR/NA *	56.9	2265	8
SCC1	CEM I 42.5 R *	-	2308	9
SCC2	CEM III/A 42.5 N-LH/HSR/NA *	-	2257	10
SCC3	CEM V/A (S-V) 32.5R-LH *	-	2198	11
SCC4	CEM I 42.5 R *	-	2187	12
SCCs0	CEM I 42.5 R *	48.6	2320	8
SCCs25	CEM I 42.5 R *	47.5	2370	8
SCCs50	CEM I 42.5 R *	46.8	2470	7
SCCs75	CEM I 42.5 R *	44.3	2520	9
SCCs100	CEM I 42.5 R *	42.4	2620	10
CP1	CEM II 52.5 R *	78.0	2493	10
CP2	CEM I 42.5 R	58.3	2359	11
HSC1	CEM I 32.5 R *	99.5	2530	6

* CEM I—portland cement; CEM II—multicomponent portland cement; CEM III—blast cement; CEM IV—pozzolanic cement; CEM V—multicomponent cement; CEM II/B (35), CEM III/A (65), CEM IV/B (55), CEM III/A (65), CEM V/A (60)—maximum content of nonclinker principal components (%), R-high-strength early cement grade, L-low early cement grade, N-grade with normal early cement strength, NA-low-alkali cement, HSR-sulfate-resistant cement, LH-with low heat of hydration.

**Table 3:** Chemical compositions of cement.

Constituent, % Mass	CEM I 42.5 R	CEM III/A 42.5 N-LH/HSR/NA	CEM V/A (S-V) 32.5R-LH	CEM I/N/SR3/NA	CEM IV/B (V) 32.5 R–LH/NA
SiO_2_	19.38	29.08	29.2	21.15	30.18
Al_2_O_3_	4.57	6.30	9.5	3.93	11.92
Fe_2_O_3_	3.59	1.37	2.8	5.14	4.72
CaO	63.78	48.82	49.3	63.34	41.95
MgO	1.38	4.36	2.4	1.28	1.72
K_2_O	0.58	0.73	0.0	0.39	1.43
Na_2_O	0.21	0.34	0.0	0.21	0.39
Eq. Na_2_O	0.59	0.82	1.3	0.47	1.33
SO_3_	3.26	2.74	2.2	2.61	2.65
Cl	0.069	0.066	0.0	0.058	0.057

**Table 4:** Overview of performed test methods.

Test ID.	Name	Parameter	Reference	Section
RCPT1	Rapid chloride penetration test: AASHTO T 277 and ASTM C1202-97	$\bar{D_{NE}}$	Equation (1), [9]	3.1
RCPT2	Rapid chloride penetration test: NT BUILD 492 (AgNO_3_ spray test applied)	$D_{T}$	Equation (2), [14]	3.2.1 and 3.2.2
NSC1	Migration in nonsteady conditions Modified NT BUILD 492 (method of fitting to a concentration curve)	$D_{migr}^{1}$	Equation (3) [48]	3.2.3 and 3.6
NSC2	Migration in nonsteady conditions Modified NT BUILD 492 (calculated based on $D_{migr}^{1}$)	$D_{dif}^{1}$	Equation (3) [48]	3.2.4
CPT	Chloride penetration test: NT BUILD 443 and ASTM C 1556	D	Equations (4) and (5) [4,5]	3.3 and 3.6
TD1	Natural diffusion with t_1_ = (method of fitting to a concentration curve)	$D^{t1}$	Equations (4) and (5)	3.4 and 3.6
TD2	Natural diffusion with t_2_ = (method of fitting to a concentration curve)	$D^{t2}$	Equations (4) and (5)	3.4 and 3.6
TDX	Thermodynamic model of migration Migration in nonsteady conditions t^1^ = 24 h and t^2^ = 48 ho	$D^{1}$	Equation (6) [41]	3.5 and 3.6

**Table 5:** Diffusion test duration of all of the test series.

Series ^1^	Test Duration (Days)		
	*t*_1_	*t*_2_	*t*_3_
1	90	180	-
2	30	60	-
3	30	60	-
4	90	180	-
5	30	-	-
6	90	180	360

^1^ Note: See Table 1 for the reference to the series and concrete mixtures.

**Table 6:** Results calculated charge *Q* for chloride migration tests RCPT1 and diffusion coefficient computed according to Equation (1) for selected concretes.

MIXTURE ID.	Calculated Charge *Q*(C)			Diffusion Coefficient According to Equation (1)–RCPT1				
				$D_{NE}\cdot 10^{-12}\;\frac{m^{2}}{s}$			$\bar{D_{NE}}\cdot 10^{-12}\;\frac{m^{2}}{s}$	σ
C6	1322	1474	1590	3.32	3.71	4.0	3.67	0.28
C7	737	718	768	1.85	1.81	1.93	1.86	0.05
C8	518	561	515	1.30	1.41	1.3	1.34	0.05
SCC1	2345	1991	2139	5.89	5.01	5.38	5.42	0.36
SCC2	250	316	297	0.63	0.79	0.75	0.72	0.07
SCC3	108	125	106	0.27	0.31	0.27	0.28	0.02
SCC4	2231	1877	2131	5.61	4.72	5.36	5.23	0.37
CP1	352	366	364	0.89	0.92	0.92	0.91	0.01
HSCC1	17.17	18.69	18.72	0.04	0.05	0.05	0.05	0.00

**Table 7:** Comparison of chloride concentration $c_{d}$ related to chloride penetration depth $x_{d}$ according to NT BUILD 492 (RCPT2).

Mixture ID	$\mathrm{Penetration}\;\mathrm{Depth}\;x_{d}(\mathrm{mm})$	$\mathrm{Chloride}\;\mathrm{Concentration}\;c_{d}$ $(\%)\;\mathrm{at}\;x_{d}\;\mathrm{Level}$
C6	9	0.38
C7	25	0.1
C8	35	0.32
SCC1	28	0.012
SCC2	9	0.2
SCC3	7	0.055
SCC4	20	0.042
CP1	10	0.55

**Table 8:** Diffusion coefficient calculated based on the standards and the thermodynamic model of migration for relating concretes.

Mix ID	Diffusion Coefficient Calculated (10^−12^ m^2^/s)							
	$\mathrm{RCPT}1\;D_{N-E}\;$ $;\;\left(\sigma \right){\;}^{**}$	$\mathrm{RCPT}2\;D_{T};\;(\partial ){\;}^{***}$	$\mathrm{NSC}1\;D_{migr}^{1}10^{-3};\;(s\left(x\right)){\;}^{*}$	$\mathrm{NSC}2\;D_{dyf}^{1};\;(s\left(x\right)){\;}^{*}$	$\mathrm{CPT}\;D;\;(s\left(x\right)){\;}^{*}$	$\mathrm{TD}1\;D^{t1};\;(s\left(x\right)){\;}^{*}$	$\mathrm{TD}2\;D^{t2};\;{\left(s\left(x\right)\right)}^{\;*}$	$\mathrm{TDX}\;D^{\;1}$
C1	-	-	-	-	-	1.36; (0.27)	1.36; (0.87)	1.36
C2	-	-	-	-	-	4.40; (0.65)	2.25; (0.83)	2.14
C3	-	-	-	-	-	1.2; (0.26)	1.2; (2.6)	0.46
C4	-	-	-	-	-	9.46; (0.41)	4.73; (0.56)	4.73
C5	-	-	-	-	-	2.25; (0.28)	2.25; (1.37)	2.25
C6	3.67; (0.28)	0.48; (±0.06)	12.5; (0.69)	12.5; (0.69)	1.20; (0.85)	4.84; (0.34)	2.42; (0.61)	4.84
C7	1.86; (0.05)	1.41; (±0.06)	130; (0.48)	130; (0.48)	2.32; (0.67)	5.88; (0.54)	4.52; (0.33)	2.27
C8	1.34; (0.05)	2.0; (±0.06)	16; (0.74)	16; (0.74)	2.96; (0.21)	3.84; (0.25)	1.92; (0.62)	1.48
SCC1	5.42; (0.36)	1.59; (±0.06)	160; (0.17)	160; (0.17)	-	4.20; (0.39)	3.23; (0.56)	3.23
SCC2	0.72; (0.07)	0.48; (±0.06)	70; (0.09)	70; (0.09)	-	2,67; (0.19)	3.47; (0.19)	2.67
SCC3	0.28; (0.02)	0.36; (±0.06)	9900; (0.00)	9900; (0.00)	-	3.65; (0.84)	3.65; (0.69)	3.65
SCC4	5.23; (0.37)	1.12; (±0.06)	3000; (0.00)	3000; (0.00)	-	3.15; (0.44)	2.56; (0.48)	1.97
SCCs0	-	-	-	-	-	0.98; (0.26)	0.98; (0.78)	0.98
SCCs25	-	-	-	-	-	1.20; (0.29)	1.07; (0.49)	1.07
SCCs50	-	-	-	-	-	1.21; (0.28)	1.21; (0.49)	1.21
SCCs75	-	-	-	-	-	1.43; (0.19)	1.20; (0.59)	1.43
SCCs100	-	-	-	-	-	3.08; (0.61)	1.54; (0.43)	1.54
CP1	0.91; (0.01)	0.54; (±0.06)	7; (0.12)	7; (0.12)	1.44; (0.11)	0.72; (0.36)	-	0.72
CP2	-	-	-	-	6.3; (1.93)	3.31; (0.28)	-	3.31
HSC1	0.05; (0.00)	-	-	-	-	0.32; (0.62)	0.32; (0.87)	0.32

* (s(x))—the value of mean squared error in brackets, ** (σ)—the value of standard deviation in brackets, *** (∂)—the value of error due to the length of the scale in brackets.

## Data Availability

The data presented in this study are available on Zenodo 2022. https://doi.org/10.5281/zenodo.7249067 (accessed on 8 December 2022) [55].

## Nomenclature

C1–C5	Ordinary concretes differing only in the type of cement used
C6–C8	Ordinary concretes used for the production of foundation blocks differ in the cement used, and an additional sealing admixture was used in C8
SCC1–SCC4	Self-compacting concretes of various compositions
SCCs0	Self-compacting concretes without the addition of ISP slag
SCCs25	Self-compacting concretes with the addition of 25% ISP slag instead of sand
SCCs50	Self-compacting concretes with the addition of 50% ISP slag instead of sand
SCCs75	Self-compacting concretes with the addition of 75% ISP slag instead of sand
SCCs100	Self-compacting concretes with the addition of 100% ISP slag instead of sand
CP1	Concrete drilled directly from the HC-500 floor slabs
CP2	Concrete drilled directly from the prestressed lintels
HSC1	High-strength self-compacting concrete
ISP	Imperial Smelting Process: a combined lead–zinc process in which oxidized concentrates are reduced with coke in a shaft furnace and the zinc fumes are collected in a lead splash condenser
RCPT1	Rapid Chloride Penetration Test accordance AASHTO T 277 and ASTM C1202-9 standard method
RCPT2	Rapid Chloride Penetration Test accordance NT BUILD 492 standard method (AgNO_3_ spray test applied)
NSC1	Migration Test in Nonsteady conditions Modified NT BUILD 492 (method of fitting to a concentration curve)
NSC2	Migration in Nonsteady conditions Modified NT BUILD 492 (calculated based on on $D_{migr}^{1}$)
CPT	Chloride penetration test accordance NT BUILD 443 and ASTM C 1556 standard method
TD1	Natural diffusion with *t*_1_ = (method of fitting to a concentration curve)
TD2	Natural diffusion with *t*_2_ = (method of fitting to a concentration curve)
TDX	Thermodynamic model of Migration in nonsteady conditions *t*_1_ and *t*_2_
$\bar{D_{NE}}$	Diffusion coefficient determined on the basis of the RCPT1 method
$D_{T}$	Diffusion coefficient determined on the basis of the RCPT2 method
$D_{migr}^{1}$	Migration coefficient determined on the basis of the NSC1 method
$D_{dif}^{1}$	Diffusion coefficient determined on the basis of the NSC2 method
D	Diffusion coefficient determined on the basis of the CPT method
$D^{t1}$	Diffusion coefficient determined on the basis of the TD1 method
$D^{t2}$	Diffusion coefficient determined on the basis of the TD2 method
$D^{1}$	Diffusion coefficient determined on the basis of the TDX method
Note: Detailed nomenclature related to equations is given below the respective equation.	
